# Nature of action of Sitagliptin, the dipeptidyl peptidase-IV inhibitor in diabetic animals

**DOI:** 10.4103/0253-7613.68425

**Published:** 2010-08

**Authors:** Joseph A. Davis, Shuchita Singh, Sachin Sethi, Subhasis Roy, Shivani Mittra, Geetavani Rayasam, Vinay Bansal, Jitendra Sattigeri, Abhijit Ray

**Affiliations:** Department of Pharmacology, New Drug Discovery Research, Ranbaxy Research Laboratories, Gurgaon, Haryana, India; 1 Department of Medicinal Chemistry, New Drug Discovery Research, Ranbaxy Research Laboratories, Gurgaon, Haryana, India

**Keywords:** Dipeptidyl peptidase-IV, fast binding inhibitor, sitagliptin, type 2 diabetes mellitus, vildagliptin

## Abstract

**Objective:**

The aim of this study was to evaluate the dipeptidyl peptidase-IV (DPP-IV) inhibitor sitagliptin with respect to mode of inhibition and its *in vivo* duration of inhibition and efficacy in type 2 diabetes animal model.

**Materials and Methods:**

DPP-IV enzyme assay was carried out in human plasma (10 μL) or human recombinant enzyme (10 ng) using H-Gly-Pro-AMC as a substrate. The competitive nature was estimated by plotting IC_50_ values measured at different substrate concentrations on the Y axis and substrate concentration on the X axis. The tight binding nature was estimated by plotting IC_50_ values measured at different plasma volumes on the Y axis and plasma volumes on the X axis. Fast binding kinetics was assessed by progressive curves at different inhibitor concentrations in the DPP-IV assay. The reversibility of the inhibitor was assessed by a dissociation study of the DPP-IV-sitagliptin complex. Durations of DPP-IV inhibition and efficacy were shown in ob/ob mice dosed at 10 mg/kg, p.o.

**Results:**

Sitagliptin is a competitive, reversible, fast and tight binding DPP-IV inhibitor. In ob/ob mice, 10 mg/kg, (p.o.) showed a long duration of inhibition of > 70% at 8 h. The duration was translated into long duration of efficacy (~ 35% glucose excursion at 8 h) in the same model and the effect was comparable to vildagliptin.

**Conclusion:**

The DPP-IV inhibitor sitagliptin behaves as a competitive, tight, and fast binding inhibitor. Sitagliptin differs mechanistically from vildagliptin and exhibits comparable efficacy to that of latter. The finding may give an understanding to develop-second generation DPP-IV inhibitors with desired kinetic profiles.

## Introduction

Dipeptidyl peptidase-IV (DPP-IV) is a serine protease cleaving dipeptides from the N-terminal end of polypeptides with L-proline or L-alanine at the penultimate position and occurs as a soluble as well as membrane-bound form in many tissues and body fluids.[1] DPP-IV either alters or abolishes many of the circulating biological peptides including glucose-dependent insulinotropic polypeptide (GIP).[2–4] However, DPP-IV has high affinity for GLP-1 and inactivates it, thereby reducing its half-life (< 2 min).[5,6]

GLP-1 is an incretin secreted by L-cells of intestine in a glucose-dependent manner. Several approaches have been tried to elevate active GLP-1 of which DPP-IV inhibitors have proven to be the promising agents to treat type 2 diabetes mellitus (T2DM).[6] While the DPP-IV inhibitor, sitagliptin (Januvia^®^), was approved worldwide as a first-in-class drug, saxagliptin (ONGLYZA^®^) was recently approved by FDA and vildagliptin (Galvus^®^) by EU market. DPP-IV inhibitors enhance active GLP-1 mediating various anti-diabetic effects such as stimulation of insulin secretion, inhibition of glucagon, promoting β cell preservation and delayed gastric emptying,[7] and are devoid of side effects such as nausea and vomiting.[4] Sitagliptin (MK-0431) is an orally active, potent, selective DPP-IV inhibitor with excellent oral bioavailability and efficacious in animal models.[8] However, details of kinetic mechanisms of the DPP-IV inhibition of sitagliptin have not been reported in contrast to other DPP-IV inhibitors vildagliptin[9] (LAF-237), BI 1356,[10] and NVP-DPP728[11] which have been developed as competitive and reversible inhibitors. The mechanistic aspects of enzyme inhibition are crucial in understanding the pharmacological properties and development of inhibitors. In this context, the present study was carried out to investigate the mechanistic aspects of sitagliptin inhibition of DPP-IV activity and its correlation to *in vivo* duration of inhibition and glucose-lowering effect.

## Materials and Methods

### Chemicals

Compounds MK-0431, LAF-237 and NVP-DPP728 were synthesized in the Department of Medicinal Chemistry, Ranbaxy Research Laboratories, as described.[8,9,12] H-Glycyl-Prolyl-7-amino-4-methylcoumarin (H-Gly-Pro-AMC) was purchased from Bachem AG (Bubendorf, Switzerland). 7-amino-4-methylcoumarin (AMC) was purchased from Sigma Chemical Co., (St. Louis, MO., USA). Insulin and GLP-1 (active), rat/mouse ELISA kits were purchased from LINCO Research Inc., (St. Charles, MO., USA). The human recombinant DPP-IV was purchased from R&D Systems (Minneapolis, MN, USA). The citrated human plasma was procured from the local blood bank.

### Animals

The ob/ob mice (8-10 weeks old, either sex) were procured from in-house animal breeding facility, provided standard laboratory chow (Harlan Teklad, Oxon, UK) and water *ad libitum* and maintained on a 12 h day/night schedule. All experiments were conducted according to the Guidelines of Experimental Animal Care issued by the Committee for Purpose of Control and Supervision of Experiments on Animals (CPCSEA) regulated by the Government of India.

### DPP-IV Enzyme Assay

Assay was carried out using either 10 *μ*l human plasma or 10 ng human recombinant DPP-IV as described.[9] Briefly, the enzyme was incubated in 50 *μ*l of buffer containing 50 mM HEPES, 80 mM MgCl_2_, 140 mM NaCl and 1% BSA, pH 7.8 at 25°C using 40 *μ*M H-Gly-Pro-AMC in the presence or absence of test compound for 20 min. The reaction was arrested by adding 50 *μ*l of 25% acetic acid and the AMC release was measured using PolarStar Galaxy fluorescence plate reader (BMG LabTechnologies Ltd, Australia) at 380 nm excitation/460 nm emission respectively.

### Competitive and Tight Binding Nature of Sitagliptin

The mode of inhibition of a compound for an enzyme can be assessed by IC_50_ values versus increasing substrate concentrations plot.[13] The IC_50_ values of sitagliptin for DPP-IV inhibition were estimated at different concentrations of H-Gly-Pro-AMC (0, 50, 100, 250, 500, 1000, and 2000 *μ*M) using human recombinant DPP-IV as enzyme source and plotted against the substrate concentrations. To find the tight binding nature, the IC_50_ values of sitagliptin for DPP-IV inhibition were measured at different human plasma volumes using 40 *μ*M H-Gly-Pro-AMC as the substrate and plotted against the increasing volumes of plasma.[13]

### Kinetics of inhibition of DPP-IV by Sitagliptin

Inhibition studies were performed by the addition of enzyme to the mix, pre-incubated with substrate and various concentrations of sitagliptin (0, 5, 12.5, 25, 50, and 125 nM final) and the progress curves of AMC liberation were monitored for every 30 s for a period of 15 min using PolarStar Galaxy fluorescence plate reader (BMG LabTechnologies Ltd, Australia) at 380 nm excitation/460 nm emission.[11] All the reactions were carried out using 10 ng of human recombinant DPP-IV in the assay buffer (100 *μ*l) containing 40 *μ*M H-Gly-Pro-AMC. Blank values (substrate without enzyme) were subtracted from data.

### Dissociation Studies

The dissociation experiments were carried out as described.[11] Briefly, 10 ng human recombinant DPP-IV were incubated without inhibitor and in the presence of 500 nM sitagliptin or 50 nM vildagliptin (approximately 10-fold excess of their IC_50_) at 25°C for 60 min. The aliquots were diluted with > 100-fold excess of the substrate H-Gly-Pro-AMC in the assay buffer. The dissociation of the enzyme-inhibitor complex was monitored by substrate hydrolysis by measuring the fluorescence every 30 s for 60 min. Blanks were subtracted from the reading and the analysis carried out.

### Duration of DPP-IV Inhibition

The ob/ob mice bred in-house were fasted for 16 h and treated orally with or without DPP-IV inhibitors at 10 mg/kg. Sitagliptin, vildagliptin, and NVP-DPP728 were suspended in 0.25% carboxy methyl cellulose (vehicle). Control animals were treated with vehicle only. Each group was allocated nine animals. Blood samples (30-40 *μ*l) were collected from the retro-orbital plexus into tubes containing EDTA at 60, 120, 240, 480, and 720 min. after administration of the compounds. The samples were centrifuged at 3000 rpm for 10 min at 4°C and the plasma subjected to measurement of DPP-IV activity.

### Duration of Glucose-Lowering Effect

The ob/ob mice were grouped into nine of nine animals each (five males and four females) as follows:
Group 1: 0.25% carboxymethylcellulose (vehicle), p.o;
Group 2: Sitagliptin treatment for 1 h (10 mg/kg), p.o;
Group 3: Sitagliptin treatment for 4 h (10 mg/kg), p.o;
Group 4: Sitagliptin treatment for 8 h (10 mg/kg), p.o;
Group 5: Sitagliptin treatment for 12 h (10 mg/kg), p.o;
Group 6: Vildagliptin treatment for 1 h (10 mg/kg), p.o;
Group 7: Vildagliptin treatment for 4 h (10 mg/kg), p.o;
Group 8: Vildagliptin treatment for 8 h (10 mg/kg), p.o;
Group 9: Vildagliptin treatment for 12 h (10 mg/kg), p.o;

The DPP-IV inhibitors were administered to overnight fasted ob/ob mice for the above stated period, followed by oral glucose challenge (2 g/kg). Blood samples collected at 0, 15, 30, 60, and 120 min after glucose load were processed as described earlier and blood glucose concentrations measured using Biochemical AutoAnalyzer, Dimension AR (Dade Behring, USA). AUC_0-120_ min were calculated and the percent glucose excursion was estimated at different duration of treatment compared to vehicle.

### Statistical Analysis

Data are presented as mean ± S.E.M. Significance of the difference between the inhibition of DPP-IV treated with sitagliptin and vildagliptin in ob/ob mice was analyzed by performing Student’s *t*-test. In each group, n=9 animals. A *P* value less than 0.05 was considered statistically significant. GraphPad prism software 4.02 was employed for IC_50_ calculations (sigmoidal dose-response curve), data, and statistical analysis.

## Results

### Sitagliptin is Competitive and Tight Binding Inhibitor

**Figure 1:**
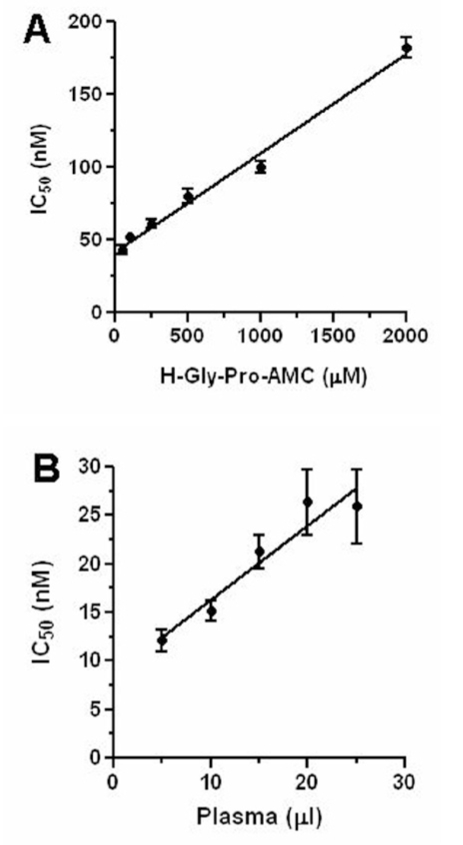
(A) Competitive nature of sitagliptin. IC_50_s determined at different substrate concentrations using 10 ng human recombinant DPP-IV enzyme plotted against increasing substrate concentrations. The values represent the mean ± S.E.M (n=3); (B) Tight binding nature of sitagliptin. IC_50_s determined at different human plasma volume plotted against increasing plasma volume (DPP-IV source). The values represent the mean ± S.E.M (n=3).

Earlier, it was found that sitagliptin is potent in inhibiting DPP-IV activity in nM range (~20 nM) and selective over various proline-specific proteases (unpublished observations). In order to characterize the nature of inhibition, IC_50_ values of sitagliptin determined at different substrate concentrations were plotted against substrate (H-Gly-Pro-AMC) concentrations [Figure 1A]. IC_50_ values of sitagliptin increased linearly with substrate concentrations indicating the competitive nature of inhibition. We next wanted to examine whether the inhibition is of tight binding in nature. Indeed, sitagliptin was found to be tight binding as their IC_50_ values increased linearly with enzyme concentration [Figure 1B]. Their intercept on the Y-axis (8.6 ± 2.5 nM) gave a good approximation of the IC_50_ value in non-tight binding conditions. Thus, sitagliptin is competitive and tight binding inhibitor of human DPP-IV.

### Kinetics of Inhibition of DPP-IV by Sitagliptin

**Figure 2:**
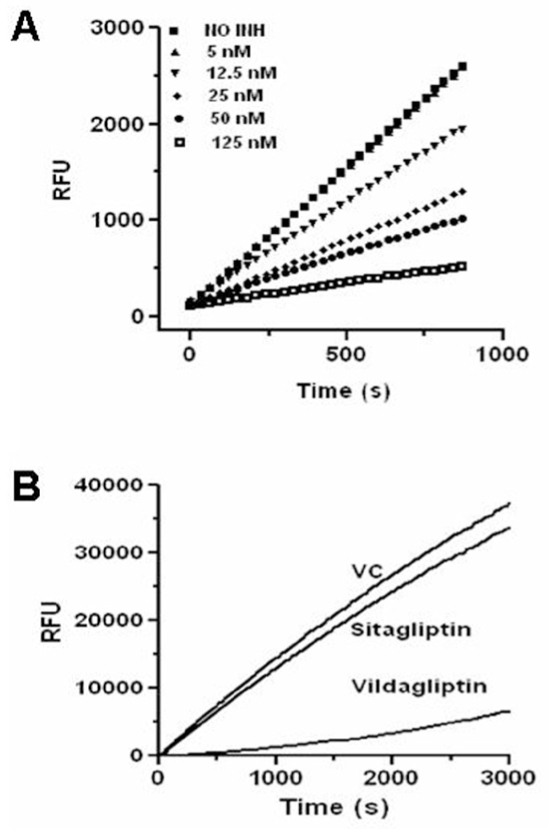
(A) Fast binding nature of sitagliptin. Inhibition studies performed by the addition of enzyme to pre-incubated mixture of substrate and various concentrations of sitagliptin (0, 5, 12.5, 25, 50 and 125 nM fi nal); (B) Sitagliptin inhibition is reversible. The human recombinant DPP-IV (10 ng) pre-incubated without (VC) or with sitagliptin (500 nM) or vildagliptin (50 nM) diluted more than 100-fold into 0.5 mM H-Gly-Pro-AMC and the DPP-IV activity measured. Both A and B represent one experiment (n=3).

Progress curves of sitagliptin at 0, 5, 12.5, 25, 50, and 125 nM were carried out to understand the mode of inhibition. The rates of the substrate (H-Gly-Pro-AMC) hydrolysis by human recombinant DPP-IV in the presence of increasing concentrations of sitagliptin was linear and the steady state was established instantaneously indicating rapid binding and classical mode of inhibition [Figure 2A].

### Sitagliptin is Reversible DPP-IV Inhibitor

In order to find the reversible nature of sitagliptin, the preformed sitagliptin/DPP-IV complex was diluted 100 fold excess of the substrate and the AMC release was monitored. As shown in Figure 2B, inhibition of DPP-IV by sitagliptin was reversible as evident from the fast relieving of the enzyme activity indicating the fast dissociation of the inhibitor. In contrast, the enzyme activity was recovered very slowly in the case of vildagliptin pre-treatment suggesting the slow dissociation of the inhibitor from the complex.

### Duration of DPP-IV Inhibition

**Figure 3:**
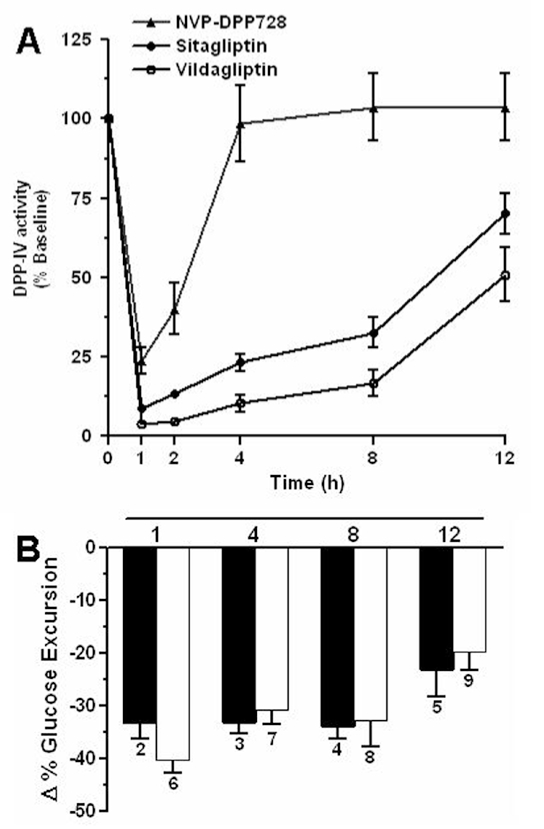
(A) Duration of DPP-IV inhibition *in vivo*. ob/ob mice were given the inhibitors at 10 mg/kg (p.o), blood collected at 0, 1, 2, 4, 8, and 12 h and analyzed for DPP-IV activity. The values represent the mean ±S.E.M (n=9). (B) Duration of glucose lowering effect *in vivo*. % AUC change in glucose in ob/ob mice pre-dosed with the inhibitors at 10 mg/kg (p.o) followed by OGTT at 1, 4, 8, and 12 h. The values represent the mean ± S.E.M (n=9). Closed bar - sitagliptin; open bar - vildagliptin; group number depicted below the bar.

The *in vivo* DPP-IV inhibitory activity of sitagliptin was evaluated in ob/ob mice. The fixed doses of both sitagliptin and vildagliptin (10 mg/kg) were chosen based on the preliminary dose titration experiments at 1, 3, and 10 mg/kg where 10 mg/kg dose exhibited maximum DPP-IV inhibition (data not shown). Administration of sitagliptin at 10 mg/kg (p.o.) resulted in 90% inhibition at 60 min and more than 70% inhibition at 8 h. For comparison, a short acting inhibitor NVP-DPP728 and long acting inhibitor vildagliptin were also included in the study in the same dosing regimen. As expected, NVP-DPP728 inhibited DPP-IV activity up to 80% for 60 min with 50% inhibition at 2 h, followed by steady recovery of enzyme activity at 4 h whereas vildagliptin inhibited more than 70% at 8 h comparable to sitagliptin [Figure 3A].

### Duration of Glucose-Lowering Effect

The 10 mg/kg dose was fixed for both sitagliptin and vildagliptin based on the DPP-IV inhibitory potency. The duration of glucose-lowering effect of sitagliptin was tested in ob/ob mice. Administration of sitagliptin and vildagliptin at 10 mg/kg (p.o.) for 0, 4, 8, and 12 h followed by the oral glucose tolerance test (OGTT) showed glucose lowering of ~ 35% at 8 h. Both inhibitors showed comparable glucose excursion with significant reduction in AUC_Glucose_ (-23 vs -20%) till 12 h [Figure 3B].

## Discussion

The incretin-based pharmacotherapies have emerged as potential anti-diabetic strategies which include DPP-IV inhibitors, GLP-1 analogs/mimetics,[4,7] and GLP-1 secretagogues.[14–16] The basis of the incretin approach was originated with the proposal of using DPP-IV inhibitors to treat T2DM.[17] DPP-IV inhibition *in vivo* should elevate both plasma active GLP-1 and insulin after a meal leading to glucose lowering.[17,18] As mentioned earlier, though there has been an in-depth kinetic analysis of various DPP-IV inhibitors,[1,10] there is not much information available for sitagliptin. The current study was carried out to characterize sitagliptin kinetically and correlate the *in vitro* profile with *in vivo* duration of DPP-IV inhibition and efficacy.

The mechanistic understanding of enzyme inhibition is crucial in designing inhibitors with superior pharmacological properties. Ideally, an enzyme inhibitor should be competitive in nature as both the inhibitor and the substrate will compete for the active site of the enzyme thereby reduce the chances of inhibiting other proline-specific proteases. Identification of DPP-IV inhibitor selective over dipeptidyl peptidase8 (DPP8), dipeptidyl peptidase9 (DPP9), and dipeptidyl peptidase II (DPP II) is important to avoid multiorgan toxicities (DPP8 and 9 non-selective) and reticulocytopenia in rats (DPP II non-selective).[19] In fact, sitagliptin behaved as a pure competitive type of inhibitor as its IC_50_ values on human plasma DPP-IV increase linearly with substrate (H-Gly-Pro-AMC) concentrations[13] and is highly selective over a variety of proline-specific proteases including DPP8, DPP89, and DPP II (Unpublished observations). Further, tight binding inhibitors are preferable from a pharmacological point of view, as the free drug is cleared from the system, whereas bound one will exert its function for a long time in the circulation. Sitagliptin was found to be a tight binding inhibitor of DPP-IV, since its IC_50_ values increase linearly as a function of enzyme concentration (Human plasma).[13] The kinetic parameters which may influence the duration of action of an inhibitor *in vivo* is its association and dissociation constants. This information is crucial in designing an inhibitor with long or short duration of action. Sitagliptin exhibited a fast binding kinetics as evident from the linear response in the presence of varying concentrations of the inhibitor. The fast binding kinetics also involves fast dissociation of the inhibitor from the inhibitor-enzyme complex in the presence of excess substrate. In fact, DPP-IV activity was recovered fast from sitagliptin-enzyme complex compared to slow recovery of DPP-IV activity from the vildagliptin-DPP-IV complex in the presence of excess substrate suggestive of fast dissociation kinetics of sitagliptin. It has been reported that vildagliptin is a slow binding inhibitor with slow dissociation constants.[1]

Since, sitagliptin exhibited fast inhibitor kinetics in contrast to slow binding kinetics of vildagliptin, it was natural to expect a differential DPP-IV inhibitory profiles of both inhibitors *in vivo*. To our surprise, sitagliptin inhibited DPP-IV to the same extent as that of vildagliptin in the same dosing regimen (10 mg/kg) (> 70% at 8 h) without significant difference. It has to be noted that the predecessor of vildagliptin, NVP-DPP728, is a less stable molecule though kinetically comparable to vildagliptin *in vitro* exhibited a shorter duration of action *in vivo*.[12] It has been established that DPP-IV inhibitors improve glucose homeostasis by augumenting both active GLP-1 and insulin in a glucose-dependent manner.[8,9,18] As expected, sitagliptin improved glucose lowering effectively till 8 h (~ 35%) comparable to vildagliptin suggesting that irrespective of the inhibitory mechanism, both inhibitors inhibit DPP-IV activity to the same extent *in vivo* and bring comparable *in vivo* efficacy. Moreover, the apparent differences in the DPP-IV inhibition kinetic profile did not reflect in the plasma levels of active GLP-1 and insulin and equally effective in augumenting both GLP-1 and insulin twofold (unpublished observations).

In conclusion, the present study demonstrates that sitagliptin is a competitive, reversible, and tight binding inhibitor. Mechanistically sitagliptin behaves as a fasting binding inhibitor with long duration of action and efficacy *in vivo*. The effect is comparable to vildagliptin, a slow binding DPP-IV inhibitor. This study may give an understanding to develop second-generation DPP-IV inhibitors with the desired kinetic mechanism.
